# Evaluation of a text supported weight maintenance programme ‘Lighten Up Plus’ following a weight reduction programme: randomised controlled trial

**DOI:** 10.1186/s12966-016-0346-1

**Published:** 2016-02-12

**Authors:** Manbinder S. Sidhu, Amanda Daley, Kate Jolly

**Affiliations:** Institute of Applied Health Research, University of Birmingham, Edgbaston, Birmingham, B15 2TT UK

**Keywords:** Text message, Obesity, Weight maintenance, Self-weighing

## Abstract

**Background:**

Many overweight people find it difficult to maintain weight loss after attending a weight reduction programme. Self-weighing and telephone support are known to be useful methods for self-monitoring for weight loss. We examined the effectiveness of an SMS-text messaging based weight maintenance programme to encourage regular self-weighing in adults who had completed a 12 week commercial weight loss programme.

**Methods:**

Randomised controlled trial of 380 obese or overweight men and women. The intervention group (*N* = 190) received a single maintenance support phone call and SMS-text based weight maintenance messages over 12 weeks to encourage regular self-weighing after completing their weight loss programme. The primary outcome was change in weight at 9 months follow up.

**Results:**

Our sample (*N* = 380) had a mean age of 47.4 years (SD 13.4), mean baseline weight and BMI of 93.1 kg (16.1) and 34.4 kg/m^2^ (5.0) respectively, as well as majority female (87.3 %) and White British (80.0 %). Using intention to treat analysis both groups regained weight at 9 months follow up; the intervention group regained an average of 1.36 kg while the control group regained 1.81 kg. Adjusting for covariates resulted in a mean difference of 0.45 kg (95 % CI −0.78, 1.67) favouring the intervention group at 9 month follow up.

**Conclusions:**

We found no evidence that an SMS based weight maintenance intervention encouraging adults to weigh themselves weekly prevented weight regain at 3 or 9 months after completing a commercial weight loss programme.

**Trial Registration:**

Current Controlled Trials ISRCTN47845106.

## Background

In the UK the rates of obesity have more than doubled in the last 25 years, and being overweight has become the norm for adults [1], but obesity is associated with a range of long-term medical conditions [2]. Health Survey for England 2009 data showed that nearly a quarter of men and women were obese [3]. To address this in the UK, 12 week commercial and NHS provided weight management programmes are frequently available and free to people who are overweight or obese. These have been shown to produce weight loss [4–7], but weight regain is common following weight loss programmes [8]. The National Institute for Health and Care Excellence (NICE) recommends maintenance programmes need to address lifestyle factors such as diet (limiting calories), increasing physical activity and reducing sedentary behaviour, as well as providing access to online material to help individuals prevent weight regain [9].

A systematic review by Dombrowski et al. [10] of long term maintenance of weight loss in obese adults identified 45 trials with 7788 individuals. Forty-two of the 45 included a formal weight loss phase prior to providing weight maintenance support. The main components during the weight maintenance phase were to address diet and physical activity. On average weight maintenance interventions lasted 12 months and had a mean of 3.2 (SD 3.19) contacts per month. At 12 months 15 studies addressing behaviour/lifestyle showed a mean difference in weight change of −1.56 kg (95 % CI −2.27 to −0.86) compared with controls. The review concluded interventions targeting diet and physical activity in combination are effective in reducing weight regain after receiving treatment for weight loss at 12 months. Notably, only three trials, all conducted in the United States, used telephone support/contact to maintain weight loss [11–13]. However, the review did not report whether weight maintenance interventions used text messages or encouraged regular self-weighing.

Research has shown that self-weighing may be a useful method of self-monitoring for both weight loss and maintenance [14–16]. The potential efficacy of self-weighing has been based on the principles of self-regulation theory [11]. Self-regulation is a process involving conscious efforts to monitor oneself, evaluate and appraise against set goals which can reinforce behaviour [17, 18]. Whilst self-weighing may be a method of supporting weight loss maintenance, people needed to be prompted to self-weigh and record their weight, so this becomes habitual [19, 20]. We hypothesised that SMS-text messages might be a low cost method to deliver regular prompts and encourage regular self-weighing.

A systematic review of the use of text messaging to achieve behaviour change in disease prevention and disease management has reported it as an effective tool for behavior change [21]. Included in this review is one trial specifically in a weight management context [22]. This small trial (*n* = 65) of intensive text-messaging (one message in the morning and one in the evening, with one to three additional reminder messages when the user thinks appropriate) during a 16-week weight loss intervention reported a significantly greater weight loss than the comparator group [22].

The aim of our study was to determine whether an SMS-text messaging based weight maintenance service to encourage self-weighing compared to a brief telephone call and leaflet of strategies to maintain weight loss differed in terms of weight change at 9 months after the end of the 12 week commercial weight-loss programme.

## Methods and procedures

### Trial design

**Fig. 1:**
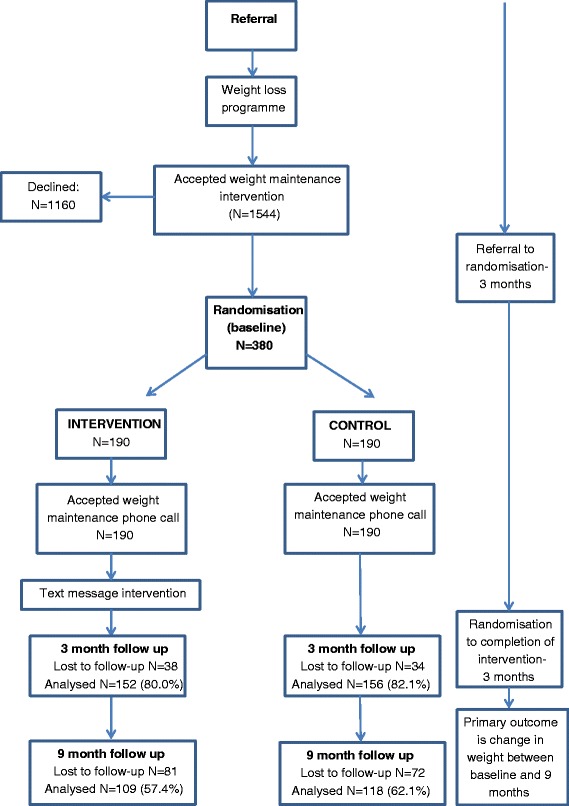
Study design and participant flow

Randomised controlled trial (RCT) with participants individually allocated to one of two weight maintenance programmes: comparator (brief maintenance support call and leaflet provided) or brief maintenance support call, leaflet plus text intervention that encouraged regular self-weighing. For trial design see Fig. 1.

### Ethics, consent and permissions

NHS ethical approval was obtained January 2013 from South Birmingham Research Ethics Committee (12/WM/0372).

### Setting and recruitment of participants

Patients registered with general practices in Birmingham, who had taken up the offer of the free 12-week Lighten Up weight management programme (Slimming World, Weight Watchers and Rosemary Conley), and had attended a minimum nine out of 12 weekly sessions were eligible. A community interest company (Gateway Family Services) co-ordinated the delivery of usual care and weight maintenance intervention via their call centre staff. The commercial weight loss organisations were contracted by the local authority who commissioned the weight management service. The commercial organisations were unaware of this trial as the programmes were provided as part of standard care and the Lighten Up Plus trial only commenced at the cessation of the free 12-week duration commercial programmes. The commercial weight loss organisations had no relationship with the research team and no input to the research.

Participants were sent a letter of invitation along with an information sheet at week nine of their weight loss programme. At baseline (end of the weight loss programme and our study baseline), call centre staff at Gateway explained the purpose of the trial and the nature of the interventions they could expect. Participants were mailed a written copy of their informed consent form. Recruitment took place between September 2013 and February 2014.

### Eligibility

Participants were eligible to take part if they were: aged 18 years or over; had a final (week 12) weight recorded or attended the Lighten Up weight management service for a minimum of nine sessions and had their weight measured within the past 2 weeks at their weight loss programme, had access to scales to weigh themselves and owned a telephone (mobile or land-line). Participants were ineligible to take part if they were: unable to understand English, pregnant, not willing to be randomised or discontinued with their weight loss programme before week nine.

### Randomisation and allocation concealment

After answering eligibility questions and obtaining verbal consent, the Gateway call centre informed participants of their allocation according to a computer generated randomisation list. A block randomisation sequence was used. The list was placed in number ordered opaque envelopes for the call centre staff to open consecutively. To ensure envelopes were used in the correct order they were dated and the identification number of the participant was recorded. Staff recorded group allocation and asked those allocated to the intervention group the nature of text message support they would like to receive, these being either continued weight loss or maintenance of current weight loss.

### Comparator group

Participants were informed that researchers were comparing two different ways of helping people to avoid weight regain. Usual care participants received a brief weight maintenance support conversation by telephone from call centre staff (for approximately 10–15 min giving lifestyle information on a balanced diet, portion control, and regular exercise) with a short leaflet with hints and tips for weight loss maintenance which was mailed at the end of their 12 week weight loss programme. The purpose of the phone call was to reinforce messages which would support the prevention of weight regain. This was followed by a further call after 3 months recapping hints and tips and completion of 3 month follow up. Call centre staff provided information only. They were not trained in delivering behavioural change interventions and had no specialist weight management skills.

### Weight maintenance intervention group: description of the service

The intervention group received the same as the comparator group as described previously. In addition, they received a 12 week duration SMS-text messaging programme to encourage the prevention of weight regain through weekly self-weighing. The intervention was developed by the university research team with input from commissioners of the weight management service. The programme was delivered by NHS Florence (FLO) who operated the automated text message system (www.getflorence.co.uk). At study baseline, call centre staff described to participants the supportive nature of text messages, the frequency of messages they would receive, that they would be prompted to self-weigh themselves weekly, the format in which they should reply their weight measurements, and helped registration with FLO. We have used the Michie et al. [23] CALO-RE taxonomy to detail the behaviour change techniques used in the intervention (Appendix 1).

#### Behavioural support

After receiving maintenance support, participants were offered text messages to encourage weight loss or maintenance through weekly self-weighing. Other behavioural change strategies included the monitoring of weight loss by another ‘person’, feedback on the outcome of the behaviour by receipt of a text response to a weight sent each week and the setting of a weight loss/maintenance target.

#### Text messages

Participants were sent an invitation text, then weekly texts asking for their weight and whether they had gained or lost/maintained their weight from the previous week. In reply they received one of three possible responses: a congratulatory message if weight was maintained/lost; advice about diet and increasing physical activity for weight gain <2 kg if this occurred for less than three successive weeks; or offer of telephone support for weight gain 3 weeks in succession. If participants did not respond to a text within 24 h a reminder text was sent. If the participant continued to regain weight they were offered referral to receive specialist support to manage their weight. Objective data, with regard to adherence to the text messaging service, was obtained from administrators of the FLO messaging system. Data provided related to response to invitation text, number of texts sent to each participant, the number of weights returned and any requests to stop receiving texts.

### Blinding

Participants were blinded to their group allocation status; hence, they were unaware which trial arm they were allocated to but informed they would receive one of two weight regain prevention strategies. Assessment at 3 months was not blinded with Gateway call centre staff collecting self-reported weight measurements. A blinded assessment was completed at 9 months follow up by staff who differed from the call centre and those providing specialist support.

### Primary outcome and assessments

The primary outcome was change in weight (kg) between the end of the commercial weight loss programme (baseline) and 9 months later (i.e. 12 months after starting the weight loss programme). See Fig. 1.

### Follow-up

Participants were followed up at 3 months from baseline by call centre staff, asking for self-reported weight, current strategies used to manage weight including frequency of self-weighing, and whether they were attending a commercial weight loss programme. Weight management strategies, including self-weighing were not assessed prior to study entry (i.e. whilst participants attended commercial weight loss programmes). However, commercial weight loss programmes encouraged weekly weighing in group sessions. Participants were asked the same questions at 9 months follow up together with objective weight data. At 9 months follow up, call centre staff contacted participants by telephone to arrange an appointment with a health trainer to collect objective weight data and record current weight management strategies. Appointments took place anytime throughout the day including weekends (0900 to 1900 h) in participant’s home, general practitioner (GP) surgeries, or designated drop in sessions at the call centre office. Participants were informed follow up appointments would last no longer than 15 min. A minimum of five attempts were made to contact each participant. Self-reported weights were taken by call centre staff for participants who were unable to be seen or contacted at 9 months follow up. Participants were given a £20 shopping voucher on completion of the 9 month objective follow up, reimbursing them for time and travel.

### Demographic information

Participants reported their date of birth, gender, ethnicity, address including postcode, weight and height (BMI) at baseline.

### Sample size

To detect a 2 kg difference in weight at the 9 month follow-up (1 year from joining Lighten Up service) between the text-intervention and usual care groups, a sample of 261 participants needed to be randomised to each group to achieve 90 % power and 5 % significance and 190 participants per group to achieve 80 % power. This estimate was based on a standard deviation of the difference in weight loss of 5.8 kg from Lighten Up data [5] and allowed for 30 % attrition at 9 months follow up. Due to a change in the initial Lighten Up service provision we curtailed recruitment when we achieved 80 % power (total of 380 participants).

### Data analysis

All analyses were conducted using SPSS version 20. Continuous variables are shown as means and standard deviations or medians and interquartile range, and categorical variables as numbers and percentages. All analyses were conducted using the intention to treat principle (ITT) and participants with missing weight data were assumed to have the mean weight change of the usual care group. The choice of imputation method was made before any analysis was undertaken. The method was selected as the most conservative approach for interpretation of within group weight change from study start, as our prior hypothesis was that the usual care group would have the most weight regain. Baseline observation carried forward is frequently used in weight loss trials, but would underestimate missing weights since most people gain weight following the cessation of a weight loss programme. Wing et al. [24] have used a method of adding 0.3 kg per month for missing weight data. Our approach is more conservative.

Within group t-tests were used to examine if each group had lost a significant amount of weight between baseline and 3 months. The difference in weight change between the groups was analysed using linear regression adjusted for baseline weight. In a sensitivity analysis we adjusted for the baseline variables weight, age, gender and ethnicity to correct for any minor imbalances. The proportion of participants in each group who achieved and maintained a 5 % weight loss since entering the Lighten Up service (beginning of weight loss programme) 12 months previously and the weight management strategies used are presented as percentages and 95 % confidence intervals or standard deviations.

## Results

### Baseline characteristics

Participant flow and characteristics have been presented in Fig. 1 and Table 1. The groups were very similar with regard to gender, age, ethnicity, employment, baseline weight, BMI, and distribution of which weight loss programme they had attended. At 3 months follow up, 152 (80.0 %) of the intervention group and 156 (82.1 %) of the control group were followed up to obtain weight data. Respectively, at 9 months follow up, 109 (57.4 %) of the intervention group and 118 (62.1 %) of the control group were followed up. Objective weights were obtained for 60.3 % (137) of those followed-up at 9 months.

**Table 1:** Participant characteristics

Variable	All participants	Intervention group	Control group
	n (%)	n (%)	n (%)
Number	380 (100)	190 (50)	190 (50)
Mean age in years (SD)	47.4 (13.4)	47.8 (13.1)	47.0 (13.7)
Weight on joining Lighten Up Service/kg (SD)	99.4 (17.0)	99.7 (17.3)	99.3 (16.6)
Weight on joining Lighten Up Plus/kg (SD)	93.1 (16.1)	93.4 (16.2)	93.0 (16.0)
Baseline BMI (SD)	34.4 (5.0)	34.5 (4.8)	34.4 (5.3)
Gender			
Male	39 (10.3)	20 (10.5)	19 (10.0)
Female	341 (87.3)	170 (89.5)	171 (90.0)
Ethnicity			
White British/ Irish/Other	304 (80.0)	152 (80.0)	152 (80.0)
Mixed Caribbean/African/other	11 (2.9)	6 (3.2)	5 (2.6)
Black Caribbean/African/Other	31 (8.2)	14 (7.4)	17 (8.9)
Asian Indian/Pakistani/Bangladeshi Other	26 (6.9)	15 (7.9)	11 (5.8)
Declined	8 (2.1)	3 (1.6)	5 (2.6)
Weight loss programme			
Rosemary Conley	48 (12.6)	23 (12.1)	25 (13.2)
Slimming World	243 (63.9)	118 (62.1)	125 (65.8)
Weight Watchers	89 (23.4)	49 (25.8)	40 (21.1)
Occupation			
Employed/Education/Carer	128 (33.7)	65 (33.2)	63 (34.2)
Not working/unemployed	27 (7.1)	16 (8.4)	11 (5.8)
Retired	28 (7.4)	14 (7.4)	14 (7.4)
Unable to code/Missing	197 (51.8)	95	102
IMD quartile			
1 (least deprived)	15 (3.9)	9 (4.7)	6 (3.2)
2	28 (7.4)	16 (8.4)	12 (6.3)
3	66 (17.4)	38 (20.0)	28 (14.7)
4	74 (19.5)	31 (16.3)	43 (22.6)
5 (most deprived)	197 (51.8)	96 (50.5)	101 (53.2)

### Primary outcome analyses

Whilst the difference in weight favoured the intervention group, there was no significant difference at 9 months between the weight change from baseline in the intervention and usual care groups (−0.46 kg, 95 % CI −1.69, 0.78). This did not change after adjusting for covariates (Table 2).

**Table 2:** Weight difference from baseline vs comparator at 3 and 9 months follow up

	Intervention	Control	Mean difference^b^	95 % CI	Adjusted mean difference^c^	95 % CI
3 months						
Crude weight difference/kg (sd)	−1.92 (4.44)	−1.76 (5.59)	−0.15	−1.28, 0.98	−0.18	−1.33, 0.98
Weight difference imputed for missing data^a^/kg (sd)	−1.90 (3.98)	−1.78 (5.06)	−0.12	−1.04, 0.79	−0.11	−1.05, 0.82
9 months						
Crude weight difference/kg (sd)	1.02 (8.23)	1.81 (7.65)	−0.80	−2.87, 1.28	−0.80	−2.80, 1.21
Weight difference imputed for missing data^a^/kg (sd)	1.36 (6.24)	1.81 (6.02)	−0.46	−1.69, 0.78	−0.45	−1.67, 0.78

^a^Missing data imputed with mean weight change for usual care group; ^b^Adjusted for baseline weight; ^c^Adjusted for baseline weight, age, gender, ethnic group; self-reported data at 3 months

There was no significant difference in self-reported weight change from baseline to 3 months between the intervention and control groups, or in the percentage of participants who lost 5 % of their body weight from the start of the Lighten Up service to follow-up a year later (118, 62.1 % vs 120, 63.2 % for the intervention and usual care groups respectively). At 3 months follow-up, both groups had lost statistically significant amounts of weight from baseline (1.9 kg (sd 4.0) in the intervention group and 1.8 kg (5.1) in the usual care group); *p* < 0.001 in each group.

### Weight management strategies

A number of weight management strategies were used in the intervention group, with 124 (82.1 %) of those followed up self-weighing at least weekly at 3 months in the intervention group compared with 112 (72.2 %) in the control group (*p* = 0.04). At 9 months the frequency of at least weekly weighing was similar in both groups (64.9 % vs 69.5 % in the intervention and usual care groups respectively) (Table 3). However, in both arms of the trial a significant proportion of participants continued to attend commercial weight loss programmes, 147 (48.0 %) at 3 months and 89 (45.7 %) at 9 months.

**Table 3:** Weight management strategies

Variable	3 months		9 months	
	n (%)		n (%)	
	Intervention	Control	Intervention	Control
Self-weighing frequency	*N* = 151	*N* = 155	*N* = 97	*N* = 105
Never	0 (0)	5 (2.6)	6 (6.2)	8 (7.6)
Once a year	2 (1.1)	0 (0)	5 (5.2)	5 (4.8)
Several times per year	2 (1.1)	10 (5.3)	10 (10.3)	6 (5.7)
Once a month	23 (12.1)	28 (14.7)	13 (13.4)	13 (12.4)
Weekly	112 (58.9)	91 (47.9)	49 (50.5)	55 (52.4)
Several times a week	8 (4.2)	16 (8.4)	9 (9.3)	14 (13.3)
Daily	4 (2.1)	5 (2.6)	5 (5.2)	4 (3.8)
Regular weight management strategies used	*N* = 151	*N* = 155	*N* = 97	*N* = 105
Planning meals	77 (40.5)	78 (41.1)	41 (43.6)	48 (47.5)
Pacing eating	59 (31.1)	51 (26.8)	33 (36.3)	23 (24.0)
Keeping a record	26 (13.7)	50 (26.3)	14 (15.4)	22 (21.8)
Portion control	102 (53.7)	103 (54.2)	45 (47.4)	52 (51.5)
Increased activity	53 (27.9)	63 (33.2)	26 (28.3)	35 (35.0)
Regular eating	129 (67.9)	128 (67.4)	67 (72.0)	72 (73.5)
Attending commercial programmes	*N* = 151	*N* = 155	*N* = 94	*N* = 105
Weight Watchers	18 (11.9)	13 (8.4)	7 (7.4)	6 (5.7)
Slimming World	58 (38.4)	46 (29.7)	27 (28.7)	36 (34.3)
Rosemary Conley	7 (4.6)	4 (2.6)	3 (3.2)	2 (1.9)
Other	0 (0)	1 (0.6)	5 (5.3)	3 (2.9)
None	68 (45.0)	91 (58.7)	52 (55.3)	58 (55.2)

### Post-hoc analyses

Given the high proportion of participants in both the intervention and usual care groups attending commercial weight management programmes where they will have received a weekly weigh-in, we undertook post-hoc analyses excluding these participants. At the 3-month follow-up the adjusted mean difference between the intervention and usual care groups was 0.16 kg (95 % CI −1.61 to 0.90). A similar picture was seen for participants not attending a commercial weight loss programme at 9-months follow-up (adjusted mean weight loss 0.02 kg; 95 % CI −2.72 to 2.28). Results only differed marginally using a completer analysis. More of the intervention group participants (64.7 %) reported at least weekly self-weighing than the usual care participants (56.0 %) at the 3 month follow-up. This difference was not maintained at 9 months.

### Adherence to the text message intervention

There was evidence of engagement with self-weighing and feedback of weights with a median of 9 (IQR 3, 13) weights texted back out of a possible 13. In total, 31 participants (16.3 %) requested to stop receiving text messages. However, from this group, the level of interaction varied before opting out; ten did not engage and left the service within the first week, five received 15 or more reminder messages but only responded to the agreement to join message, while 16 sent weight readings before opting out.

### Loss to follow up

Participants who were lost to follow-up at 9 months were younger than those followed-up (42.8 years compared to 50.5 years) and had a slightly higher BMI on starting the study (Table 4). A higher proportion of women and those of White British ethnicity were lost to follow-up compared to men and participants from minority ethnic groups. Follow-up was lower in participants who had initially attended Slimming World and Weight Watchers and higher in those who had attended Rosemary Conley.

**Table 4:** Participant characteristics of those followed-up and lost to follow-up at 9 months

Variable	Followed-up at 9 m	Lost to follow-up at 9 m
	n (%)	n (%)
Number	227 (59.7)	153 (40.3)
Mean age in years (SD)	50.5 (12.3)	42.8 (13.6)
Weight on joining Lighten Up Service/kg (SD)	98.7 (16.0)	100.5 (18.3)
Weight on joining Lighten Up Plus/kg (SD)	92.4 (15.4)	94.5 (16.8)
Baseline BMI (SD)	34.0 (4.8)	35.1 (5.4)
Weight loss during Lighten Up (SD)	6.4 (4.0)	6.2 (4.0)
Gender		
Male (%)	30 (76.9)	9 (23.1)
Female (%)	197 (57.8)	144 (42.2)
Ethnicity		
White British/ Irish/Other	176 (58.3)	126 (41.7)
Minority ethnic group/Mixed	47 (67.1)	23 (32.9)
Weight loss programme		
Rosemary Conley	33 (68.8)	15 (31.3)
Slimming World	140 (57.6)	103 (42.4)
Weight Watchers	54 (60.7)	35 (39.3)
Occupation		
Employed/Education/Carer	68 (53.1)	60 (46.8)
Not working/unemployed	14 (58.3)	10 (41.7)
Retired/sick/disabled	20 (64.5)	11 (35.5)
Unable to code/Missing	125 (63.5)	72 (36.5)
IMD quintile		
1 (least deprived)	7 (3.1)	8 (5.2)
2	18 (7.9)	11 (7.2)
3	43 (18.9)	24 (15.7)
4	52 (22.9)	22 (14.4)
5 (most deprived)	107 (47.1)	88 (57.5)

## Discussion

We found no evidence that an SMS based weight maintenance intervention significantly reduced weight at 3 or 9 months after completing a free 12 week commercial weight loss programme, although the direction of change favoured the intervention group. Participants in both study groups showed resistance to weight regain in the short term, but the effects were not maintained in the longer term. Process data from the intervention group showed good engagement with a text-message service that encouraged weekly self-weighing and recording weight. Other trials of the use of text messaging to achieve behaviour change in disease prevention and disease management have reported it as an effective tool for behavior change [25]. Two of the studies within the Cole-Lewis review investigated SMS-text messaging in weight loss [26, 27]. Both reported effective text messaging interventions with a weight loss range of 2.9–4.5 kg. Haapala and colleagues randomised 126 overweight adults to a text message or a no-contact control group [26]. After 12 months, the intervention group lost more weight than the control group (4.5 kg/m^2^ vs 1.1 kg/m^2^, *P* = .006, respectively). Most of the weight loss occurred during the first 3 months when usage of the text message programme was high. The interventions in both trials [26, 27] were more intensive than ours, with participants able to initiate contact. Additionally they were both focused on weight loss, rather than weight maintenance. Text messaging also shows promise in the support of increasing physical activity in a review of four trials (*N* = 432) [28]. A review of reviews [29] concluded than SMS-text messaging interventions for weight loss can be effective, but that there is insufficient evidence about long-term effectiveness and that larger trials and cost-effectiveness studies are required. A systematic review of long-term weight maintenance [10] suggested a benefit from support for maintenance of weight loss, provided long term support was provided of more than 24 months.

Given the positive examples described above, the design of future text-based maintenance services used may have to be altered to increase effectiveness to prevent weight regain, possibly through increased intensity and interactivity. Evidence shows interventions encompassing both diet and exercise are most successful in preventing weight gain [30]. In particular, providing specific information, for instance minutes exercising per day or regulating calorie intake [31] can better support maintenance compared to generic material. A review by Hartmann-Boyce et al. [32] aimed at identifying effective self-help strategies to support weight loss found that other than self-monitoring and giving advice about diet and physical activity, technological interaction with goal setting plus self-monitoring helped intervention participants lose more weight than controls.

There was a high level of continued attendance at commercial weight loss programmes during both the intervention phase of this study and beyond in both trial arms, with 48 % of responders still attending (10 % Weight Watchers, 34 % Slimming World, 4 % Rosemary Conley respectively) at 3 months follow up and 41 % at 9 months. This high level of continued attendance at commercial programmes may have led to mixed messages received by participants as many commercial weight loss programme group leaders discourage self-weighing between the weekly classes, as differences between home scales and those used for session weigh-ins can be de-motivating [33–36]. It also may have diluted the ability of this trial to detect a difference between trial groups since controls would have been receiving weekly weight loss support. To address this we undertook a post-hoc analysis in participants who did not report continued attendance at a commercial weight loss group. We found no difference in weight change between intervention and usual care participants in this subgroup.

Many individuals who have lost weight encounter difficultly in preventing weight regain in the medium to long term i.e. experience relapse. Limited studies have focused primarily on preventing weight regain after attending weight loss programmes. A text messaging based weight maintenance service can encourage greater self-weighing and recording of weight, but there is no evidence that it can promote behaviour change. Future research might focus on provision of personalised information about diet and/or physical activity, along with specific weekly weight-related goal setting.

Existing literature presents favourable conclusions towards the use of remote technologies in encouraging and managing weight loss. For example, a recent RCT (*N* = 70) in the United States found that their intervention group, which received a digital device to help participants record food intake (calories) and physical activity once a week supplemented with tele-coaching for 6 months, lost more weight after completing a weight loss programme compared to the control group at 12 months follow up (8.6 pounds, 95 % CI [4.9, 12.2]). As a result, remote technologies can be beneficial in the prevention of weight regain post completing a weight loss programme but may require the provision of intensive multicomponent monitoring with simultaneous personalised support [37].

### Strengths and limitations

The strengths of the study are that it was a pragmatic RCT conducted within the context of an easy to deliver weight management service. A large sample size was recruited and the follow-up rate at 3 months was 81.1 %. In the intention to treat analyses, we used a conservative method to impute missing weight data. Objective data were available to determine the response to the text messages and we were able to quantify each participant’s level of engagement with the programme. Our sample was from a large ethnically diverse population, although men made up only 10 % of the sample which reflects the participants attending commercial weight loss providers in the UK [6, 7] but is relatively lower than recruitment rates of men in weight loss trials i.e. approximately 30 % [38, 39]. There were no systematic differences in characteristics between those who were lost to follow up and completed follow up. There are limitations to this trial. We under-recruited to achieve 90 % power (intended recruitment target was 522 participants) but we nevertheless achieved 80 % power. Loss to follow up at 9 month follow up was high (40 %) which is similar to other trials of weight loss interventions [40, 41], although there were no demographic differences between those who were followed up and those who were not. Given that the sample size included allowance for 30 % loss to follow up at 9 months but 40 % were lost to follow up it is possible this trial was marginally under powered. It also appears that intervention contamination has occurred whereby about 48–52 % of control participants were also weighing weekly.

Finally, a number of participants in our trial continued to attend commercial weight loss providers which may have limited the ability of the trial to detect an effect.

## Conclusion

A text-based weight maintenance intervention that encouraged weekly self-weighing was not significantly better to prevent weight regain compared to usual care. Given the increasing prevalence of obesity internationally, easy to deliver weight management services are ever more important to prevent weight regain; yet, the application of tele-health requires re-consideration, in particular how to provide continued behavioural support.

## Appendix

**Table 5:** Behaviour change techniques used in the SMS-text based Lighten UP Plus intervention

Name of behavioural change technique	Description
Provide information on consequences of behaviour in general	Information about the relationship between the behaviour and its possible or likely consequences in the general case, usually based on epidemiological data, and not personalised for the individual
	*Example text message*
	*Your weight has increased a little bit. Eating more and exercising less can lead to you gaining weight. Plan your meals and walk instead of taking the car.*
Goal setting (outcome)	The person is encouraged to set a general goal that can be achieved by behavioural means but is not defined in terms of behaviour (e.g. to reduce blood pressure or lose/maintain weight), as opposed to a goal based on changing behaviour as such.
	*Example text message*
	*Remember to watch your weight! It’s ok to have a treat occasionally but not too often. Try to spend less time sitting and more moving next week.*
Prompt review of behavioural goals	Involves a review or analysis of the extent to which previously set behavioural goals (e.g. take more exercise next week) were achieved. In most cases this will follow previous goal setting and an attempt to act on those goals, followed by a revision or readjustment of goals, and/ or means to attain them.
	*Example text message*
	*Hi, this is FLO. Please send your weight reading in kgs. Have you gained weight this week? (e.g. WT 86.5 No)*
Prompt rewards contingent on effort or progress towards behaviour	Involves the person using praise or rewards for attempts at achieving a behavioural goal. This might include efforts made towards achieving the behaviour, or progress made in preparatory steps towards the behaviour, but not merely participation in intervention.
	*Example text message*
	*Weigh to go!! You’ve not gained weight! You set yourself a goal and achieved it. Brilliant!*
Prompt self-monitoring of behavioural outcome	The person is asked to keep a record of specified measures expected to be influenced by the behaviour change, e.g. blood pressure, blood glucose, weight loss, physical fitness.
	*Example text message*
	*Hi, it’s FLO. You will shortly receive a text asking about your weight. Start your message with “WT” then “Your weight in kgs” and “Yes or No” e.g. WT 80.5 Yes”*
Provide feedback on performance	This involves providing the participant with data about their own recorded behaviour or commenting on a person’s behavioural performance
	*Example text message*
	*Brilliant! It takes a lot of willpower to lose weight. Keep going and you will reach your target!*
